# Early Fluid is Less Fluid: Comparing Early Versus Late Resuscitation in Severely Injured Trauma Patients

**DOI:** 10.21203/rs.3.rs-3409172/v1

**Published:** 2023-10-11

**Authors:** Catherine E. Beni, Saman Arbabi, Bryce R.H. Robinson, Grant E. O’Keefe

**Affiliations:** University of Washington; Harborview Medical Center; Harborview Medical Center; Harborview Medical Center

**Keywords:** trauma, hemorrhagic shock, resuscitation, crystalloid, ICU

## Abstract

**Background:**

We aimed to characterize the temporal trends of crystalloid resuscitation in severely injured trauma patients after intensive care unit (ICU) admission. Using 500 mL/hr of crystalloid in the first 6 hours of ICU admission to distinguish early versus late resuscitation, we hypothesized early resuscitation was associated with less volume by 48 hours and better outcomes compared with late resuscitation.

**Methods:**

We performed a retrospective review of the trauma registry of a high-volume level 1 academic trauma center to examine adult trauma patients admitted to the ICU (2016–2019) with: with initial serum lactate ≥ 4 mmol/dL, elevated lactate (≥ 2 mmol/L) at ICU admission, and lactate normalization within 48 hours. We analyzed patient and injury characteristics, and the first 48 hours of ICU course. The primary outcome was ICU length of stay (LOS); secondary outcomes included ventilator days, acute kidney injury (AKI), and in-hospital death. We compared subjects who received early resuscitation to those received late resuscitation using unadjusted methods and multivariable regression models.

**Results:**

We analyzed 333 subjects. The late resuscitation group received less volume over the first 24 hours, but surpassed the early group by 48 hours (5.5 vs 4.1L, p ≤ 0.001). The late group had longer ICU LOS (9 vs 5 days, p ≤ 0.001) and ventilator days (5 vs 2 days, p ≤ 0.001), and higher incidence of AKI (38% vs 11%, p ≤ 0.001). On multivariable regression, late resuscitation remained associated with longer ICU LOS and ventilator days, and higher odds of AKI after adjusting for important confounders.

**Conclusions:**

After hemostasis, crystalloid can play an important role in restoration of organ perfusion. Delaying resuscitation is associated with both receipt of higher volumes of crystalloid by 48 hours and worse outcomes compared to early resuscitation. Judicious crystalloid given early in ICU admission could improve outcomes in the severely injured.

## Background

**Figure 1:**
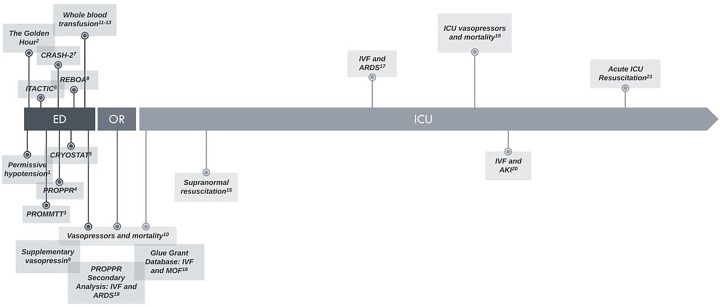
Timeline of Clinical Studies in Trauma Resuscitation

With the introduction of damage control surgery, balanced blood product transfusion, prehospital blood product administration, and, more recently, early administration of whole blood, trauma resuscitation has changed considerably in the last few decades.^1,2,11–13,3–10^ Clinical trials have primarily concentrated on resuscitation prior to hemostasis, with less attention on the period of resuscitation after intensive care unit (ICU) admission (Figure 1).^14^ Observational data indicate that larger volumes of intravenous (IV) crystalloid are associated with worse outcomes, including multiorgan failure and longer ICU length-of-stay (LOS).^15–20^ In light of increasing data regarding the adverse effects of excessive crystalloid, substantially less crystalloid is used in current resuscitation practices than in the past several decades. In an effort to better characterize resuscitation practices after ICU admission, we recently reported that higher volumes of crystalloid were associated with worse outcomes.^21^ We additionally noted that half of the subjects received 500 mL/hr crystalloid infusions more than 6 hours after ICU admission and, on subsequent analyses, this delay in resuscitation was associated with worse outcomes.

With this knowledge, we sought to better understand the association between timing of crystalloid resuscitation following ICU admission and outcomes. This study had two aims: first, to describe the time course of crystalloid resuscitation between ICU admission and lactate normalization in a more contemporary cohort of trauma patients; second, to determine differences between subjects who received earlier crystalloid resuscitation from those receiving later resuscitation. For the second aim, we used at least 500 mL/hr of crystalloid given before or after 6 hours from ICU admission to distinguish early resuscitation from late resuscitation. Our primary hypotheses were that early resuscitation would be associated with 1) less crystalloid by 48 hours after ICU admission and 2) shorter ICU LOS. Our secondary hypotheses were that early resuscitation was associated with shorter time on the ventilator and lower rates of acute kidney injury (AKI) than late resuscitation. In order to better understand differences between these two groups, we also examined the reasons for crystalloid infusions. Taken together, this information will be helpful in developing guidelines for resuscitation following hemorrhage control.

## Methods

### Additional Analysis of 2012–2015 Study Data

In reanalyzing our cohort of subjects admitted from 2012–2015, half received ≥ 500 mL crystalloid infused over a period of 15–60 minutes more than 6 hours after arriving in the ICU. We therefore chose 6 hours as a threshold to distinguish early from late resuscitation. We defined *early resuscitation* as at least 500 mL of crystalloid (normal saline or lactated Ringer’s) infused over 15–60 minutes within 6 hours of ICU admission. *Late resuscitation* was defined as receiving at least 500 mL of crystalloid over 15–60 minutes between 6 and 48 hours after ICU admission.

Utilizing the 2012–2015 data, we determined a minimum sample size of 64 per group was required to identify a 3-day difference in ICU LOS with 80% power. Descriptions of the 2012–2015 cohort may be found in the supplementary material. The remainder of our analyses here focus on the 2016–2019 cohort.

### Study Cohort and Data Collection

Our study was conducted in accordance with the STROBE guidelines for observational studies.^22^ We analyzed data from our institutional trauma registry linked with electronic health record data for patients admitted to our ICU from January 1, 2016 to December 31, 2019. This study was approved by our Institutional Review Board under protocol STUDY00009850 (Most recent update - December 2020), and the need for informed consent was waived. All procedures were followed in accordance with the Helsinki Declaration.

Subjects were included if: 1) age ≥ 18; 2) initial lactate ≥ 4 mmol/L; 3) lactate ≥ 2 mmol/L at ICU admission; and 4) administered IV crystalloid in the time between ICU admission and serum lactate reaching ≤ 2 mmol/L or death.^21^ We excluded subjects with isolated severe traumatic brain injuries (head abbreviated injury scale [AIS] ≥ 3, all other AIS ≤ 1) or who were alive but had no documented lactate ≤ 2 mmol/L within the first 48 hours of ICU admission. *Acute ICU resuscitation* refers to the period between ICU admission and lactate normalization.

We obtained demographics, comorbidities, injury characteristics, and data from the first 48 hours of ICU admission, including vital signs, interventions, laboratory values, and outcomes. The primary outcome of interest was ICU-free days in 28. Secondary outcomes were ventilator-free days in 28, acute respiratory distress syndrome (ARDS), AKI, onset of AKI from ICU admission, in-hospital death, and discharge home. AKI was assigned using the creatinine-based criteria from the Kidney Disease Improving Global Outcomes (KDIGO) group guidelines, while ARDS was defined according to the National Trauma Data Standard.^23,24^

For each subject, we obtained hourly crystalloid administered over both acute ICU resuscitation and the first 48 hours of ICU admission. To address our first aim of understanding the temporal patterns of crystalloid use, we classified subjects into one of four categories by whether they received early or late resuscitation: 1) no instance of crystalloid ≥ 500 mL given over 15–60 minutes (*minimal resuscitation*), 2) only early resuscitation (*early resuscitation*), 3) only late resuscitation (late resuscitation), and 4) both early and late resuscitation (*extended resuscitation*).

To assign indications for crystalloid administration, we collected vital signs (heart rate [HR]), mean arterial pressure [MAP]), urine output (UOP), laboratory values (lactate, creatinine [Cr], blood urea nitrogen [BUN]), vasopressor usage, and operative intervention in the two hours prior to the first 500 mL bolus. Indications were classified as follows: 1) hypotension if MAP < 65 mmHg or a decrease in MAP by 5 mmHg; 2) tachycardia if HR > 120 bpm or an increase in HR by 20 bpm; 3) oliguria if UOP < 0.5 mL/kg/hour; 4) renal dysfunction if Cr > 1.5 mg/dL, BUN:Cr > 20 with Cr > 1 mg/dL, or AKI; 5) elevated lactate if lactate > 2 mmol/L; 6) operative intervention if the subject returned from the operating room; 7) vasopressors if any of norepinephrine, epinephrine, phenylephrine, or dobutamine were started or the dose was increased – excluding isolated phenylephrine given to achieve an elevated MAP goal, such as for spinal cord injury. We first identified whether a bolus was given in the setting of hypotension, and then allowed for multiple classifications.

### Data Presentation and Statistical Analyses

We described continuous variables using medians and interquartile range (IQR) and categorical variables using count and proportion. For our first objective, we present summary statistics for the entire cohort followed by the four resuscitation groups.

To investigate the associations between resuscitation timing, crystalloid volume, and outcomes, we compared the early and late resuscitation groups. We compared patient and injury characteristics, vital signs, ICU resuscitation, and outcomes between the two groups; using Mann-Whitney-U test and Chi-squared test for continuous and categorical variables respectively.

We then used backwards stepwise multivariable regression analyses to measure the relationships between resuscitation timing and outcomes while adjusting for important confounders; specifically, we used linear regression models for ICU LOS and duration of mechanical ventilation, and logistic regression models for AKI. We limited our linear regression analyses to survivors as there was no censoring and the competing risk of death was infrequent. Our models used demographics, comorbidities, injury characteristics, care prior to ICU admission, and care during acute ICU resuscitation as covariates. Linear regression results were reported as coefficients (β) with 95% confidence intervals (CI) while logistic regression results were reported as adjusted odds ratios (aORs) with 95% CI.

We performed two sensitivity analyses for our regression models in which replaced volume given during acute ICU resuscitation with either total volume or number of 500 mL boluses given in the first 48 hours of ICU admission. As our institution receives many patients in transfer from other hospitals, we also performed subgroup analyses excluding patients transferred from other institutions.

All data analyses were conducted using Python (Python v3.0, Python Software Foundation, https://www.python.org/) and the packages statsmodels, Pandas, SciPy, and NumPy.^25–29^

## Results

### Description of Entire Cohort and Characteristics of the Resuscitation Groups

**Figure 2:**
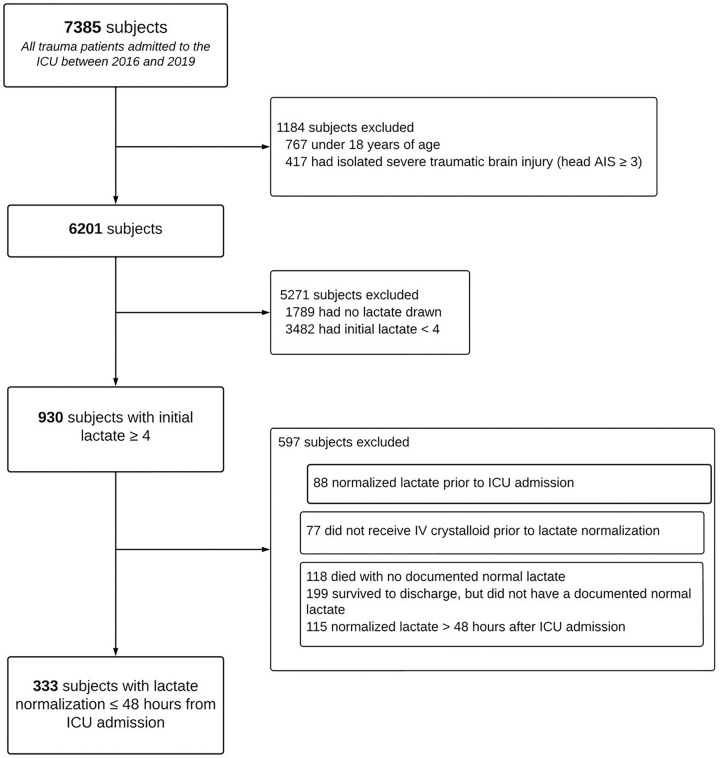
Patient Selection

A total of 333 patients were included (Figure 2). Demographics, comorbidities, injury characteristics, care prior to ICU admission, and outcomes are shown in Table 1. Subjects were typically male, middle-aged, and suffering blunt trauma. Prior to ICU admission, subjects received 2 (0, 6) units of packed red blood cells (PRBCs), 2 (0, 5) units of fresh frozen plasma (FFP), and 0 (0, 1) units of platelets; 37% were taken to the operating room. Whole blood was not available during the study period.

The median time to lactate normalization was 12 (6, 25) hours and subjects received a median 2.2 (1, 4.4) L of crystalloid. A total of 1084 resuscitative boluses were given, with hypotension (50%), oliguria without hypotension (31%), and elevated lactate without hypotension (31%) being the most common indications. Approximately one third of subjects were taken to the operating room during this period. The most common reason for operative intervention was extremity fixation (53; 16%), while 26 subjects (8%) underwent operative intervention for new or recurrent hemorrhage. Subjects had a median ICU LOS of 6 (4, 13) days and were mechanically ventilated for 4 (2, 8) days. AKI occurred in 89 subjects (27%) while 40 subjects (12%) died prior to discharge.

Patient characteristics, pre-ICU care, acute ICU resuscitation, and outcomes for the four resuscitation groups are presented in Table 2. Approximately half of the subjects were in the extended resuscitation group (47%), while the remainder were evenly distributed amongst the other three groups. Demographics and comorbidities were similar between all four groups. The extended resuscitation group was more severely injured and received more operative interventions, vasoactive medications, blood products, and crystalloid during acute ICU resuscitation. In contrast, the minimal resuscitation group was less severely injured and required lower rates of operative intervention, infrequent vasopressors, and the least amount of blood products and crystalloid to normalize lactate.

### Resuscitation Timing, Crystalloid Volume, and Outcomes

Here, we focus on comparing the early resuscitation group and the late resuscitation group (Table 2). The two groups had similar injury characteristics based upon their AIS and ISS scores. Prior to ICU admission, the late resuscitation group received more blood products; otherwise, the groups were similar. Upon ICU arrival, the two groups had similar blood lactate concentrations. The early resuscitation group reached a normal blood lactate concentration by 9 (6, 19) hours while the late group achieved a normal blood lactate by 13 (5, 25) hours. Blood product and vasopressor use during this period was similar in the two groups.

Comparing the early resus. group to late resus. group, * indicates p < 0.05, ** indicates p < 0.01,*** indicates p ≤ 0.001

**Figure 3:**
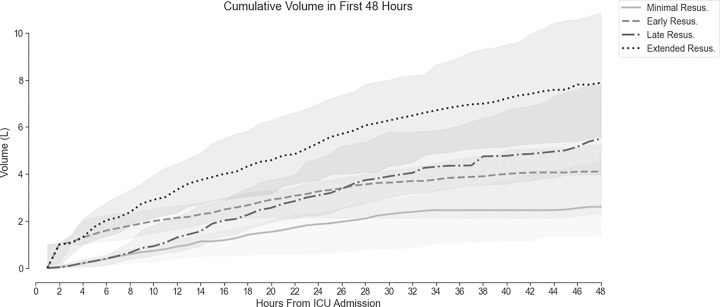
Cumulative Volume in ICU by Resuscitation Group

Figure 3 shows the median and IQR of the cumulative volume given over the first 48 hours of ICU admission. At 24 hours, subjects in both groups received similar volumes (3.1 L vs 3.3 L). By 48 hours, subjects in the late group had received more crystalloid than subjects in the early group (5.5 L vs 4.1 L, p ≤ 0.001).

Next, we looked at the indications for initial crystalloid resuscitation and whether they differed between these groups. Subjects in both groups received roughly half of their first boluses in the setting of hypotension: 45% for the early resuscitation group and 47% for the late resuscitation group. Vasopressors dose changes prior to a bolus were rare, occurring in 2% of the early group and 7% of the late group. Outside of hypotension, the early resuscitation group tended to receive boluses in the setting of elevated lactate (60% vs 25%) or upon return to the ICU from the operating room (14% vs 3%) compared with the late resuscitation group. The late resuscitation group received more fluid in the setting of tachycardia (25% vs 6%) or oliguria (53% vs 3%) compared with the early resuscitation group. Crystalloid was rarely given in the setting of biochemical renal dysfunction (0% in the early group vs 6% in the late group).

Subjects in the late resuscitation group had a longer ICU LOS than the early resuscitation group (9 vs 5 days, p ≤ 0.001). After adjusting for important covariates, late resuscitation remained associated with longer ICU LOS (β 6.49, 95% CI [3.11, 9.87], p ≤ 0.001). Similarly, for the secondary outcomes, the late resuscitation group had a longer duration of mechanical ventilation (5 vs 2 days, p ≤ 0.001), and a higher incidence of AKI (38% vs 11%, p ≤ 0.001). After adjusting for covariates, the duration of mechanical ventilation was longer (β 6.02, 95% CI [2.6, 9.44], p ≤ 0.001), and risk of AKI was higher (aOR 7.99, 95% CI [2.56, 25.03], p ≤ 0.001) in the late resuscitation group (Table 3).

For our two sensitivity analyses in which we replaced crystalloid volume during acute ICU resuscitation as an independent variable with either volume or boluses over the first 48 hours of ICU admission, we found delayed resuscitation remained associated with longer ICU LOS, increased duration of mechanical ventilation, and higher risk of AKI. Similarly, when transfer patients were excluded from our regression models, we continued to see an association between late resuscitation and longer ICU LOS, increased duration of mechanical ventilation, and higher risk of AKI. The results of our sensitivity analyses are provided in the supplementary material.

## Discussion

Using data from our previous study, we applied a 6-hour threshold to distinguish early from late resuscitation following arrival to the intensive care unit, defining four resuscitation groups: 1) a minimal resuscitation group, who were less severely injured and required little product, vasopressors, crystalloid, or operative intervention after ICU admission; 2) an early resuscitation group, who were moderately injured and received most of their crystalloid resuscitation early in ICU admission; 3) a late resuscitation group, who were similarly injured to the early resuscitation group and received their crystalloid later in ICU admission; and 4) an extended resuscitation group, who were the most severely injured and required the most resuscitative interventions.

In subjects presenting to the ICU with elevated blood lactate, we hypothesized early resuscitation would be associated with lower volumes of crystalloid and improved outcomes compared to late resuscitation. Aside from pre-ICU blood and ICU crystalloid, the early resuscitation group and the late resuscitation group received similar care both prior to and in the ICU, including having similar hyperlactatemia on ICU arrival. After receiving just under 2 L of crystalloid in the first 6 hours of ICU admission, subjects in the early resuscitation group plateaued in their resuscitative needs. In contrast, subjects in the late resuscitation group received minimal volume in the first 6 hours, yet their crystalloid requirements exceeded those in the early bolus group by 48 hours. Excluding hypotension (the incidences of which were similar), the two groups differed in their indications for crystalloid: hyperlactatemia for the early resuscitation group, and oliguria and tachycardia for the late resuscitation group. The late resuscitation group ultimately had worse outcomes, with longer ICU LOS, longer time on the ventilator, and higher risk of AKI. Our observations are consistent with the clinical application of evidence regarding the harmful effects of excessive crystalloid administration.^30–33^ Yet, while the data indicates large volumes of crystalloid can be harmful, subjects in previous reports received much higher volumes than our study (8–15 L in the first 24 hours). Our findings suggest an overly-restrictive or delayed approach to crystalloid use after hemostasis may also be harmful.^16–18^ Further, the early resuscitation group actually received more volume prior to lactate normalization than the late resuscitation group (1.7 L vs 1 L). As the approach to crystalloid use in trauma has changed over the years and considerably less volume is being given during ICU resuscitation, our data suggests the timing of crystalloid may play a greater role in determining outcomes than volume alone.

Our study has important limitations. Perhaps most important is that the early and late resuscitation groups differ in unmeasured ways. For example, factors that led to deterioration in the late bolus group, may themselves lead to worse outcomes. However, by all measures available, the two groups were similar over the 6 hours following ICU admission apart from the timing of crystalloid administration. This limitation can only be addressed by a randomized clinical trial. Next, determining indications for bolus administration required arbitrary selection of thresholds and therefore may over- or under-report some indications. Nevertheless, the general observation that there were differences in the clinical factors potentially related to crystalloid administration between the two groups is important and may help identify opportunities for earlier resuscitation. Overall, our findings suggest that the approach to crystalloid administration in our ICU is not standardized. It is likely that similar variability exists in many other ICUs.34–36 Because blood lactate is not measured at pre-specified intervals and only when requested by the clinical team, we may be either failing to recognize ongoing shock, particularly in the subjects in the delayed resuscitation group, or overestimating the time to lactate normalization. We controlled for this through sensitivity analyses utilizing a fixed time point of 48 hours, leading to similar results. Limiting our study to those subjects who had a documented normal blood lactate within 48 hours necessarily excluded many patients. Excluded patients likely differed from those included by being less severely injured and not deemed to require subsequent lactate measurements, and it is possible that more patients would fit into the group who did not require additional crystalloid resuscitation. However, this would not impact our comparisons between the early and late resuscitation groups. Finally, as the competing risk of death was infrequent, we chose to present ICU LOS and ventilator days rather than ICU-free or ventilator-free days to avoid creation of a composite outcome that may complicate interpretation. To enable comparability to other studies, our data on ICU-free and ventilator-free days are available in the SUPPLEMENATARY MATERIAL. After including deceased subjects, we found the late resuscitation regression coefficients for ICU-free days (β −4.7, 95% CI [−7.3, −2.2]) and ventilator-free days (β −3.9, 95% CI [−6.6, −1.2]) did not change our results.

## Conclusions

Once hemorrhage has been controlled, the priority shifts to restoring organ perfusion, where crystalloid still plays an important role. Giving 1–2 L of IV crystalloid in the first 6 hours of ICU admission to trauma patients with hyperlactatemia appears to be safe. Delaying resuscitation is associated with both receipt of higher volumes of crystalloid by 48 hours and worse outcomes compared to early resuscitation. Judicious use of crystalloid early in ICU admission could improve outcomes in the severely injured.

## Figures and Tables

**Table 1:** Subject Characteristics and Acute ICU Resuscitation

*Subject Characteristics*	Subjects 2016-2019 (n = 333)
Age (years)	41 (28, 55)
Male	257 (77%)
Obesity	92 (28%)
Diabetes	27 (8%)
Smoker	68 (20%)
CHF	7 (2%)
CKD	0 (0%)
Blunt trauma	266 (80%)
AIS	
Head ≥ 3	111 (33%)
Chest ≥ 3	189 (57%)
Abdomen ≥ 3	99 (30%)
Spine ≥ 3	47 (14%)
Lower extremity ≥ 3	156 (47%)
ISS	29 (17, 41)
*Pre-ICU Care*	
Transfer	73 (22%)
Initial lactate (mmol/L)	5.8 (4.6, 7.8)
RBCs transfused (units)	2 (0, 6)
FFP transfused (units)	2 (0, 5)
Platelets transfused (units)	0 (0, 1)
To OR from ED	124 (37%)
*Acute ICU Resuscitation*	
Lactate at ICU admission (mmol/L)	4.1 (3, 5.2)
Received PRBCs	89 (27%)
Received FFP	69 (21 %)
Received platelets	62 (19%)
Operative intervention	91 (27%)
Vasopressors used	85 (26%)
Volume of IVF (L)	2.2 (1, 4.4)
Duration of acute ICU resuscitation (hours)	12 (6, 25)
*Outcomes*	
ICU length of stay (days)	6 (4, 13)
Duration of mechanical ventilation (days)	4 (2, 8)
ARDS	18 (5%)
AKI	89 (27%)
AKI onset from ICU admission (hours)	11 (1, 24)
Discharge home	165 (50%)
Deceased	40 (12%)

**Table 2:** Subject Characteristics, Acute ICU Resuscitation, and Outcomes by Bolus Group

	Min. Resus. (n = 46)	Early Resus. (n = 64)	Late Resus. (n = 68)	Extended Resus. (n = 155)
*Patient Characteristics*				
Age (years)	41 (26, 57)	43 (32, 58)	38 (27, 54)	43 (28, 53)
Male	36 (78%)	49 (77%)	51 (75%)	121 (78%)
AIS				
Head ≥ 3	11 (24%)	25 (39%)	19 (28%)	56 (36%)
Chest ≥ 3	24 (52%)	30 (47%)	39 (57%)	96 (62%)
Abdomen ≥ 3	6 (13%)	12 (19%)	23 (34%)	58 (37%)
Spine ≥ 3	4 (9%)	8 (12%)	10 (15%)	25 (16%)
Lower extremity ≥ 3	15 (33%)	25 (39%)	37 (54%)	79 (51%)
Blunt trauma	31 (67%)	50 (78%)	55 (81%)	130 (84%)
ISS	22 (17, 34)	24 (17, 35)	29 (17, 43)	29 (22, 43)
*Pre-ICU Care*				
Transfer	10 (22%)	12 (19%)	20 (29%)	31 (20%)
Initial lactate (mmol/L)	5.3 (4.4, 6.8)	5.8 (4.6, 8.5)	6.1 (4.8, 7.7)	5.9 (4.7, 8)
RBCs transfused (units)	2 (0, 4)	1 (0, 4)	3 (0, 7)**	3 (0, 6)
FFP transfused (units)	1.5 (0, 4)	1 (0, 3.2)	3 (0, 8)**	3 (0, 5)
Platelets transfused (units)	0 (0, 1)	0 (0, 0)	0(0, 1)***	0(0, 1)
To OR from ED	17 (37%)	16 (25%)	25 (37%)	66 (43%)
*Acute ICU Resuscitation*				
Lactate at ICU admission (mmol/L)	3.7 (2.4, 4.7)	4.2 (3.3, 5.4)	3.8 (2.7, 5)	4.2 (3.1, 5.4)
RBCs transfused (units)	0 (0, 0)	0 (0, 0.2)	0 (0, 1)	0 (0, 1)
FFP transfused (units)	0 (0, 0)	0 (0, 0)	0 (0, 0)	0 (0, 1)
Platelets transfused (units)	0 (0, 0)	0 (0, 0)	0 (0, 0.2)	0 (0, 0)
Operative intervention	8 (17%)	16 (25%)	21 (31%)	46 (30%)
Vasopressors used	5 (11%)	10 (16%)	16 (24%)	54 (35%)
Volume of IVF (L)	0.6 (0.1, 1.3)	1.7 (1.3, 2.6)	1.1 (0.3, 2.7)**	3.7 (2.1, 6.6)
Duration of acute ICU resuscitation (hours)	7 (5, 13)	9 (6, 19)	13 (5, 25)	17 (8, 28)
*Outcomes*				
ICU length of stay (days)	5 (3, 7)	5 (3, 8)	9 (5, 18)***	7 (4, 14)
Duration of mechanical ventilation (days)	2 (1, 4)	2 (1, 6)	5 (3, 11)***	5 (2, 10)
ARDS	1 (2%)	2 (3%)	5 (7%)	10 (6%)
AKI	5 (11%)	7 (11%)	26 (38%)***	51 (33%)
AKI onset from ICU admission (hours)	22 (1, 44)	16 (11, 34)	18 (11, 33)	12 (5, 21)
Discharge home	32 (70%)	36 (56%)	33 (49%)	64 (41%)
Deceased	2 (4%)	7 (11%)	7 (10%)	24 (15%)

Comparing the early resus. group to late resus. group

* indicates p < 0.05

** indicates p < 0.01

*** indicates p ≤ 0.001

**Table 3:** Regression Analyses

Independent Variable	Coeff/OR	95% CI	P
*ICU length of stay (days)*			
Chest AIS ≥ 3	5.28	[1.9, 8.65]	0.002
RBCs before ICU admission (units)	0.34	[0.04, 0.63]	0.026
Vasopressor use during acute ICU resuscitation	9.64	[0.31, 18.96]	0.043
Late resus.	6.04	[2.59, 9.5]	≤ 0.001
*Duration of mechanical ventilation (days)*			
Chest AIS ≥ 3	4.19	[0.69, 7.69]	0.019
Late resus.	5.56	[2.05, 9.06]	0.002
AKI			
Age (years)	1.04	[1.01, 1.07]	0.008
Initial lactate (mmol/L)	1.19	[1.07, 1.34]	0.002
Platelets during acute ICU resuscitation (units)	2.23	[1.09, 4.53]	0.027
Late resus.	9.58	[2.95, 31.19]	≤ 0.001
Volume during acute ICU resuscitation (L)	1.29	[1.03, 1.63]	0.03

## Data Availability

The datasets generated and/or analysed during the current study are not publicly available due to identifiable patient data, but de-identified subsets are available from the corresponding author on reasonable request.

## Abbreviations

AKI: acute kidney injury
ARDS: acute respiratory distress syndrome
ICU: intensive care unit
IV: intravenous
LOS: length of stay

## References

[1] Bickell WH, Wall MJ, Pepe PE, Martin RR, Ginger VF, Allen MK, Immediate versus delayed fluid resuscitation for hypotensive patients with penetrating torso injuries. N Engl J Med. 1994;331:1105–9. 7935634 10.1056/NEJM199410273311701

[2] Blow O, Magliore L, Claridge JA, Butler K, Young J. The golden hour and the silver day: Detection and correction of occult hypoperfusion within 24 hours improves outcome from major trauma. J Trauma - Inj Infect Crit Care. 1999;47(5):964–9. 10.1097/00005373-199911000-00028 10568731

[3] Holcomb JB, Fox EE, Wade CE. The PRospective observational multicenter major trauma transfusion (PROMMTT) study: Comparative Effectiveness of a Time-Varying Treatment with Competing Risks. JAMA. 2013;148(2):127–36. 10.1001/2013.jamasurg.387 PMC3740072 23560283

[4] Holcomb JB, Tilley BC, Baraniuk S, Fox EE, Wade CE, Podbielski JM, Transfusion of plasma, platelets, and red blood cells in a 1:1:1 vs a 1:1:2 ratio and mortality in patients with severe trauma: The PROPPR randomized clinical trial. JAMA - J Am Med Assoc. 2015;313(5):471–82. 10.1001/jama.2015.12 PMC4374744 25647203

[5] Curry N, Rourke C, Davenport R, Beer S, Pankhurst L, Deary A, Early cryoprecipitate for major haemorrhage in trauma: A randomised controlled feasibility trial. Br J Anaesth. 2015;115(1):76–83. 25991760 10.1093/bja/aev134

[6] Baksaas-Aasen K, Gall LS, Stensballe J, Juffermans NP, Curry N, Maegele M, Viscoelastic haemostatic assay augmented protocols for major trauma haemorrhage (ITACTIC): a randomized, controlled trial. Intensive Care Med [Internet]. 2021;47(1):49–59. Available from: 10.1007/s00134-020-06266-1 33048195 PMC7550843

[7] Roberts I, Shakur H, Coats T, Hunt B, Balogun E, Barnetson L, The CRASH-2 trial: A randomised controlled trial and economic evaluation of the effects of tranexamic acid on death, vascular occlusive events and transfusion requirement in bleeding trauma patients. Health Technol Assess (Rockv). 2013;17(10):1–80. 10.3310/hta17100 PMC4780956 23477634

[8] Du Bose JJ, Scalea TM, Brenner M, Skiada D, Inaba K, Cannon J, The AAST prospective Aortic Occlusion for Resuscitation in Trauma and Acute Care Surgery (AORTA) registry: Data on contemporary utilization and outcomes of aortic occlusion and resuscitative balloon occlusion of the aorta (REBOA). J Trauma Acute Care Surg. 2016;81(3):409–19. 27050883 10.1097/TA.0000000000001079

[9] Sims CA, Holena D, Kim P, Pascual J, Smith B, Martin N, Effect of Low-Dose Supplementation of Arginine Vasopressin on Need for Blood Product Transfusions in Patients with Trauma and Hemorrhagic Shock: A Randomized Clinical Trial. JAMA Surg. 2019;154(11):994–1003. 31461138 10.1001/jamasurg.2019.2884 PMC6714462

[10] Sperry JL, Minei JP, Frankel HL, West MA, Harbrecht BG, Moore EE, Early use of vasopressors after injury: Caution before constriction. J Trauma Acute Care Surg. 2008;64(1):9–14. 10.1097/TA.0b013e31815dd029 18188092

[11] Cotton BA, Podbielski J, Camp E, Welch T, Del Junco D, Bai Y, A randomized controlled pilot trial of modified whole blood versus component therapy in severely injured patients requiring large volume transfusions. Ann Surg. 2013;258(4):527–32. 23979267 10.1097/SLA.0b013e3182a4ffa0

[12] Spinella PC, Pidcoke HF, Strandenes G, Hervig T, Fisher A, Jenkins D, Whole blood for hemostatic resuscitation of major bleeding. Transfusion. 2016;56(April):S190–202. 27100756 10.1111/trf.13491

[13] Weymouth W, Long B, Koyfman A, Winckler C. Whole Blood in Trauma: A Review for Emergency Clinicians. J Emerg Med. 2019;56(5):491–8. 30904380 10.1016/j.jemermed.2019.01.024

[14] Braasch MC, Turco LM, Cole E, Brohi K, Winfield RD. The Evolution of Initial-Hemostatic Resuscitation and the Void of Post-Hemostatic Resuscitation. J Trauma Acute Care Surg. 2020;89(3):597–601. 32826738 10.1097/TA.0000000000002576

[15] Shoemaker WC, Appel PL, Kram HB, Waxman K, Lee TS. Prospective trial of supranormal values of survivors as therapeutic goals in high-risk surgical patients. Chest. 1988;94(6):1176–86. 3191758 10.1378/chest.94.6.1176

[16] Kasotakis G, Sideris A, Yang Y, de Moya M, Alam H, King D, Aggressive Early Crystalloid Resuscitation adversely affects Outcomes in Adult Blunt Trauma Patients: An Analysis of the Glue Grant Database. J Trauma Acute Care Surg. 2010;36(3):490–9. 10.1097/TA.0b013e3182826e13 PMC3984883 23609270

[17] Plurad D, Martin M, Green D, Salim A, Inaba K, Belzberg H, The decreasing incidence of late posttraumatic acute respiratory distress syndrome: the potential role of lung protective ventilation and conservative transfusion practice. J Trauma Acute Care Surg [Internet]. 2007;63(1):1–7. Available from: internal-pdf://215.197.187.79/Plurad - ARDS and 48hr Fluids - Trauma - 2007.pdf 10.1097/TA.0b013e318068b1ed 17622861

[18] Robinson BRH, Cohen MJ, Holcomb JB, Pritts TA, Gomaa D, Fox EE, Risk Factors for the Development of Acute Respiratory Distress Syndrome Following Hemorrhage. Shock [Internet]. 2018;50(3):258–64. Available from: internal-pdf://201.218.48.125/ARDS PROPPR Shock2018.pdf internal-pdf://2706896572/ARDS PROPPR Shock20181.pdf internal-pdf://0561717988/ARDS PROPPR Shock20182.pdf 29194339 10.1097/SHK.0000000000001073 PMC5976504

[19] Plurad DS, Talving P, Lam L, Inaba K, Green D, Demetriades D. Early vasopressor use in critical injury is associated with mortality independent from volume status. J Trauma Acute Care Surg. 2011;71(3):565–72. 10.1097/TA.0b013e3182213d52 21908995

[20] Hatton GE, Du RE, Wei S, Harvin JA, Finkel KW, Wade CE, Positive Fluid Balance and Association with Post-Traumatic Acute Kidney Injury. J Am Coll Surg. 2020;230(2):190–199.e1. 31733328 10.1016/j.jamcollsurg.2019.10.009 PMC7220831

[21] Beni CE, Arbabi S, Robinson BRH, O’Keefe GE. Acute intensive care unit resuscitation of severely injured trauma patients: Do we need a new strategy? J Trauma Acute Care Surg. 2021;91(6):1010–7. 34347741 10.1097/TA.0000000000003373 PMC9009679

[22] Guidelines STROBE - PLOS - 2007.pdf.

[23] Kellum JA, Lameire N, Aspelin P, Barsoum RS, Burdmann EA, Goldstein SL, Kidney disease: Improving global outcomes (KDIGO) acute kidney injury work group. KDIGO clinical practice guideline for acute kidney injury. Kidney Int Suppl. 2012;2(1):1–138.

[24] National Trauma Data Standard: Data Dictionary [Internet]. American College of Surgeons. 2020. Available from: https://www.facs.org/-/media/files/quality-programs/trauma/ntdb/ntds/data-dictionaries/ntds_data_dictionary_2021.ashx

[25] Van Rossum G.; Drake FL. Python 3 Reference Manual. Scotts Valley, CA. 2009.

[26] Virtanen P, Gommers R, Oliphant TE, Haberland M, Reddy T, Cournapeau D, SciPy 1.0: fundamental algorithms for scientific computing in Python. Nat Methods. 2020;17:261–72. 32015543 10.1038/s41592-019-0686-2 PMC7056644

[27] Seabold S, Perktold J. Statsmodels: Econometric and Statistical Modeling with Python. Proc 9th Python Sci Conf. 2010;

[28] McKinney W. Data Structures for Statistical Computing in Python. Proc 9th Python Sci Conf. 2010;

[29] Oliphant T, Millma J k. A guide to NumPy. Trelgol Publishing. 2006.

[30] Malbrain ML, Marik PE, Witters I, Cordemans C, Kirkpatrick AW, Roberts DJ, Fluid overload, de-resuscitation, and outcomes in critically ill or injured patients: a systematic review with suggestions for clinical practice. Anaesthesiol Intensive Ther [Internet]. 2014/11/30. 2014;46(5):361–80. Available from: internal-pdf://68.56.7.39/Malbrain Fluid Overload and De-resuscitation 2.pdf internal-pdf://2442186215/Malbrain Anaesthesiology Intensive Therapy 201.pdf internal-pdf://2426206510/Malbrain Fluid Overload and De-resuscitation R.pdf internal-pdf://135951218 25432556 10.5603/AIT.2014.0060

[31] Cordemans C, De Laet I, Van Regenmortel N, Schoonheydt K, Dits H, Huber W, Fluid management in critically ill patients: the role of extravascular lung water, abdominal hypertension, capillary leak, and fluid balance. Ann Intensive Care [Internet]. 2012/08/10. 2012;2(Suppl 1 Diagnosis and management of intra-abdominal hyperten):S1. Available from: https://www.ncbi.nlm.nih.gov/pubmed/22873410 22873410 10.1186/2110-5820-2-S1-S1 PMC3390304

[32] Salahuddin N, Sammani M, Hamdan A, Joseph M, Al-Nemary Y, Alquaiz R, Fluid overload is an independent risk factor for acute kidney injury in critically Ill patients: results of a cohort study. BMC Nephrol. 2017;18(1):45. 28143505 10.1186/s12882-017-0460-6 PMC5286805

[33] Barmparas G, Liou D, Lee D, Fierro N, Bloom M, Ley E, Impact of positive fluid balance on critically ill surgical patients: a prospective observational study. J Crit Care [Internet]. 2014;29(6):936–41. Available from: https://www.ncbi.nlm.nih.gov/pubmed/25085510 25085510 10.1016/j.jcrc.2014.06.023

[34] Sucher JF, Moore FA, Todd SR, Sailors RM, McKinley BA. Computerized clinical decision support: A technology to implement and validate evidence based guidelines. J Trauma - Inj Infect Crit Care. 2008;64(2):520–37. 10.1097/TA.0b013e3181601812 18301226

[35] McKinley BA, Kozar RA, Cocanour CS, Valdivia A, Sailors RM, Ware DN, Normal versus Supranormal Oxygen Delivery Goals in Shock Resuscitation: The Response Is the Same. J Trauma [Internet]. 2002;53(5):825–32. Available from: internal-pdf://83.153.198.83/McKinley O2 Delivery Goals Resus Trauma 2002.pdf 12435930 10.1097/00005373-200211000-00004

[36] Santora RJ, Moore FA. Monitoring trauma and intensive care unit resuscitation with tissue hemoglobin oxygen saturation. Crit Care [Internet]. 2010/01/09. 2009;13 Suppl 5:S10. Available from: internal-pdf://183.158.33.181/Santora TICU Resus SkMusO2 Crit Care 2009.pdf 19951382 10.1186/cc8008 PMC2786112

